# Tanshinones from *Salvia miltiorrhiza* alleviate ulcerative colitis via reprogramming the gut microbiota-metabolite axis: Tanshinones alleviate UC via microbiota-metabolite axis

**DOI:** 10.3724/abbs.2026054

**Published:** 2026-03-31

**Authors:** Zhe Liu, Chan Hui, Guochao Zhang, Haicheng Yang, Yi Wang, Yaqian Shi, Chao Wang, Yanfei Liu, Xia Gao, Yuting Wen

**Affiliations:** 1 Department of Pathology The Ninth Hospital of Xi’an Xi’an 710054 China; 2 Department of Clinical Laboratory The Ninth Hospital of Xi’an Xi’an 710054 China; 3 Department of General Surgery Tangdu Hospital The Fourth Military Medical University Xi’an 710038 China; 4 Chinese People’s Liberation Army 94162 Military Hospital Xi’an 710600 China; 5 Department of Pathology The Affiliated Children’s Hospital of Xi’an Jiaotong University Xi’an 710003 China; 6 Department of Experimental Surgery Tangdu Hospital The Fourth Military Medical University Xi’an 710038 China

**Keywords:** *Salvia miltiorrhiza*, tanshinone, ulcerative colitis, gut microbiota, metabolic reprogramming

## Abstract

The anti-inflammatory properties of the traditional herb *Salvia miltiorrhiza* Bunge are well-established, yet its precise mechanism of action in ulcerative colitis (UC) remains unclear. Herein, we evaluate the therapeutic potential of four major tanshinones–tanshinone IIA (Tan IIA), miltirone, neocryptotanshinone, and dihydrotanshinone I–in a murine dextran sulfate sodium (DSS)-induced colitis model. Our results show that tanshinones effectively alleviate disease severity, suppress systemic and local inflammation, and restore intestinal barrier integrity. Integrated multi-omics analysis reveals that the therapeutic efficacy originates from a comprehensive reprogramming of the gut microbiota-metabolite axis. Specifically, tanshinones reverse colitis-associated dysbiosis and rectify metabolic disturbances in linoleic acid metabolism, bile acid biosynthesis, and amino acid utilization. Correlation network analysis identifies key functional modules linking beneficial microbes ( *e*. *g*., *Akkermansia*) to anti-inflammatory lipid mediators and associating pathobionts ( *e*. *g*., *Desulfovibrio*) with disrupted bile acid metabolism. Notably, supplementation with *Akkermansia muciniphila* synergizes with Tan IIA to amplify barrier restoration and metabolic normalization. Our findings establish that tanshinones ameliorate UC through microbiota-driven metabolic reprogramming, wherein the restructured microbial community actively shapes a therapeutic metabolic output. This work elucidates a metabolite-mediated mechanism of action and positions tanshinones as promising microbiome-targeting therapeutics for inflammatory bowel disease.

## Introduction

Ulcerative colitis (UC) is a chronic and relapsing inflammatory bowel disease (IBD) characterized by abdominal pain, bloody diarrhea, and weight loss [ 1, 2] . While biological therapies targeting pro-inflammatory cytokines have revolutionized treatment, their variable efficacy and secondary failure highlight an urgent need for novel therapeutic strategies [ 3, 4] .

The pathogenesis of UC involves a complex interplay of genetic susceptibility, immune dysregulation, and environmental factors [ 5– 7] . Among these, dysbiosis of the gut microbiota is recognized as a pivotal contributor [ 7, 8] . Patients with UC exhibit a distinct loss of microbial diversity, an expansion of pro-inflammatory pathobionts ( *e*. *g*., *Proteobacteria* and *Enterobacteriaceae*), and a reduction in beneficial commensals ( *e*. *g*., *Adlercreutzia*) [ 9– 12] . Importantly, gut dysbiosis in UC leads to a profound reprogramming of the gut metabolome, characterized by disrupted production of key metabolites such as short-chain fatty acids (SCFAs), bile acids (BAs), and tryptophan derivatives [ 8, 12– 14] . These metabolic alterations are not merely bystanders but active drivers of intestinal inflammation, barrier dysfunction, and immune imbalance [15]. Consequently, therapeutic strategies capable of restoring microbial ecological balance and metabolic homeostasis represent a promising avenue for UC intervention.

*Salvia miltiorrhiza* Bunge (Danshen), a renowned traditional herbal medicine, has recently demonstrated efficacy in ameliorating dextran sulfate sodium (DSS)-induced colitis in mice [16], an effect attributed to the anti-inflammatory properties of its lipophilic component tanshinones, including tanshinone IIA and cryptotanshinone [ 17– 20] . However, despite these documented direct anti-inflammatory effects, it remains unknown whether tanshinones exert their therapeutic benefits by remodeling the gut microbiota and inducing beneficial metabolic reprogramming to counteract the pathological state in UC.

**Figure 1:**
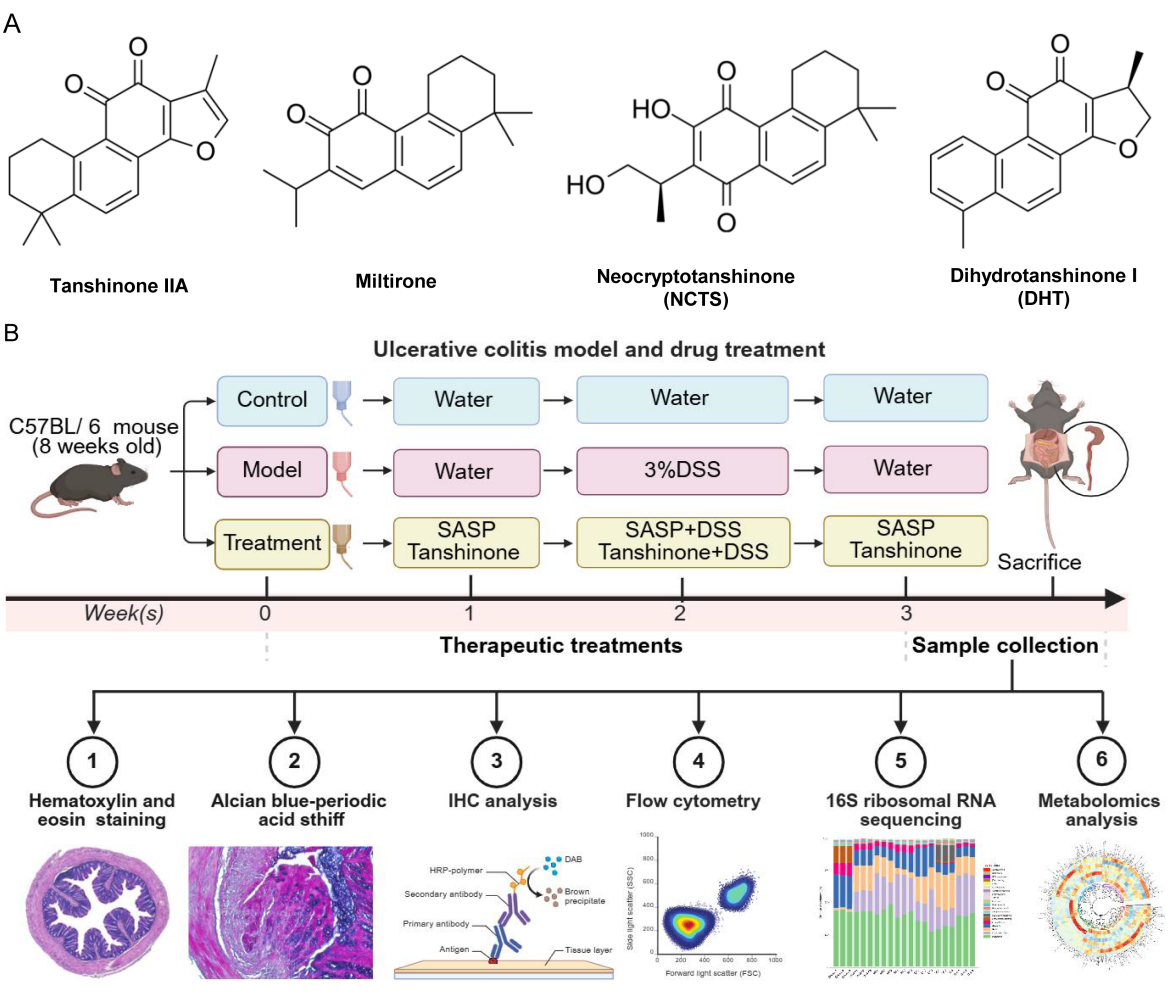
Figure 1 Schematic overview of the experimental design (A) Chemical structures of the four liposoluble tanshinones from Salvia miltiorrhiza investigated in this study. (B) Timeline of the DSS-induced colitis model and therapeutic intervention. After one week of acclimatization, mice received daily gavages of sulfasalazine (SASP, positive control) or one of the four tanshinones from week 1. Colitis was induced by administering 3% DSS in drinking water during week 2. Treatments continued until the end of the experiment on week 3 (Day 21), when samples were collected for histopathological, immunological, and multi-omics analyses to assess therapeutic efficacy and elucidate the underlying mechanisms.

To address this knowledge gap, we investigated the effects of four major liposoluble tanshinones ( Figure 1A)–tanshinone IIA (Tan IIA), miltirone, neocryptotanshinone (NCTS), and dihydrotanshinone I (DHT)–on DSS-induced murine colitis. By employing an integrated multi-omics approach combining 16S rRNA sequencing and untargeted metabolomics, we aimed to elucidate the precise mechanisms, with a specific focus on their capacity to reshape microbial communities and reprogram host-microbiota co-metabolism. Our findings provide novel insights into the microbiota-metabolite axis underlying the anti-UC effects of tanshinones, underscoring the therapeutic potential of metabolic reprogramming in IBD, and supporting its development as a microbiome-targeted therapeutic agent.

## Materials and Methods

### Reagents and materials

DSS (36–50 kDa, 160110) was purchased from MP Biomedicals (Santa Ana, USA). The tanshinone IIA (HY-N0135), miltirone (HY-N1951), Neocryptotanshinone (HY-119720), dihydrotanshinone I (HY-N0360), and sulfasalazine (HY-14655) were purchased from MedChemExpress (Monmouth Junction, USA). *Akkermansia muciniphila* ( *M*. *Akk*, BNCC341917) was obtained from BNCC (Xinyang, China). A commercial multiplex bead-based immunoassay (RayPlex Mouse Inflammation Bead Array 1) was sourced from RayBiotech (Peachtree Corners, USA). An Alcian Blue/Periodic Acid-Schiff (AB-PAS) kit (RS3790) was purchased from G-CLONE Biotech (Beijing, China). Primary antibodies against the following proteins were used for both immunohistochemistry and immunoblotting: zonula occludens-1 (ZO-1, AF5145; Affinity Biosciences, Cincinnati, USA), occludin (DF7504; Affinity Biosciences), mucin 1 (MUC1, FNab05427; Finetest, Wuhan, China), and mucin4 (MUC4, 680431; ZEN-BIOSCIENCE, Chengdu, China) for the evaluation of intestinal barrier integrity; interleukin-1β (IL-1β, GB11113; Servicebio, Wuhan, China) and IL-6 (GB11117; Servicebio) for the detection of pro-inflammatory cytokines. The β-actin (AF7018) were obtained from Affinity Biosciences.

### Study design

The overall study design was presented in Figure 1. To comprehensively evaluate the therapeutic effects of the four liposoluble tanshinones from *Salvia miltiorrhiza* on a DSS-induced UC mouse model, we employed an integrated approach combining histological staining, 16S rRNA sequencing and metabolomics. This multi-omics strategy allowed to investigate the potential mechanisms of action.

### Animal experiment

Female C57BL/6 mice (8 weeks old, 18–20 g) were purchased from the Animal Research Institute of Air Force Medical University (Xi’an, China) and housed under specific pathogen-free (SPF) conditions. All mice were confirmed to be SPF. The animals were housed under controlled temperature conditions (24°C ± 1°C) with a 12 h light/dark cycle. The experimental procedures were conducted in strict compliance with the regulations on the Administration of Affairs Concerning Experimental Animals and were approved by the Biomedical Ethics Committee of Xi’an Ninth Hospital (approval No. 2025110).

### Experimental design and therapeutic intervention

To evaluate and compare the therapeutic efficacy of the four liposoluble tanshinones, a DSS-induced murine colitis model was established. The experimental timeline is schematically illustrated in Figure 1B. Following acclimatization, mice were randomly assigned to seven groups ( *n*  = 5 per group): (1) Control (water+vehicle); (2) Model (DSS + vehicle); (3) Positive control (DSS+sulfasalazine] 100 mg/kg/day); (4–7) tanshinone groups (DSS + Tan IIA, miltirone, NCTS, or DHT, 20 mg/kg/day).

All groups received their respective daily oral gavage treatments starting from week 1. Acute colitis was induced during week 2 by supplementing the drinking water with 3% (w/v) DSS for all groups except the Control group, while oral treatments continued. At the start of week 3 (Day 15), DSS was withdrawn, but the therapeutic interventions were maintained. Body weight, stool consistency, and the presence of fecal occult blood were monitored daily to calculate the disease activity index (DAI) as described in the previous study [21]. Any mouse exhibiting >20% weight loss was to be euthanized per welfare standards; no mice in this cohort reached this endpoint. After the modeling period, all mice were euthanized. Colons were collected for length measurement, histological and immunohistochemical analysis. Colonic fecal contents were harvested and immediately frozen at –80°C for 16S ribosomal RNA (rRNA) gene sequencing and untargeted metabolomic profiling.

### Determination of growth kinetics

To characterize the growth dynamics of *M*. *Akk*, a single colony was picked from a freshly activated blood agar plate and inoculated into 10 mL of fluid thioglycolate (FT) medium. The culture was incubated overnight at 37°C under anaerobic conditions with shaking at 180 rpm. Subsequently, this pre-culture was transferred into fresh FT medium at a 1:1000 inoculation ratio, which was defined as basal value (0 h). Samples were collected at 2, 4, 6, 8, 12, 16, 24, 48, 72, and 96 h post-inoculation. Each sample underwent ten-fold serial dilutions. From appropriate dilutions, 10 μL aliquots were spotted onto designated sectors of blood agar plates. After incubation at 37°C under anaerobic conditions for an appropriate period until colonies reached a countable size, colonies on plates containing between 10 and 50 colonies were enumerated. The colony-forming units per milliliter (CFU/mL) of the original sample were calculated. A growth curve was plotted with GraphPad Prism software (version 8.4.3, GraphPad Software San Diego, USA) using cultivation time (hours) as the X-axis and CFU/mL as the Y-axis.

### Drug intervention assay

To investigate the effects of tanshinones on the growth of *M*. *Akk*, a drug intervention assay was performed. First, a freshly activated bacterial culture was inoculated into FT medium at a 1:1000 ratio and incubated for 24 h at 37°C under anaerobic conditions with shaking, allowing the bacteria to reach the late logarithmic growth phase. The initial viable cell count (CFU/mL) was determined at this point. Subsequently, four tanshinone compounds–Tan IIA, miltirone, NCTS, and DHT-were individually added to separate culture aliquots to achieve final concentrations of 400 μM, 200 μM, 100 μM, 50 μM, 25 μM, and 12 μM. After vortex mixing, the samples were incubated under static anaerobic conditions at 37°C. Samples were collected at 1, 2, 3, 4, and 5 days post-treatment. The viable bacterial count (CFU/mL) in each sample was determined using the plate colony counting method described above to assess the inhibitory or promotive effects of the different drug concentrations on bacterial growth over time.

### Evaluation of the therapeutic efficacy after Tan IIA and *M*. *Akk* treatment

A follow-up experiment was designed to validate the individual and combined effects of critical Tan IIA and the key bacterium *M*. *Akk*. Mice were randomly assigned to seven groups ( *n* = 5 per group): (1) Control (water + vehicle); (2) Model (DSS + vehicle); (3) Tan IIA + DSS (DSS + Tan IIA 20 mg/kg/day); (4) Tan IIA; (5) *M*. *Akk* (0.5 × 10 ^8^ CFU/day); (6) Tan IIA + *M*. *Akk*; and (7) Tan IIA + *M*. *Akk*  + DSS.

The experimental timeline mirrored that of the above experiment. Daily gavage (pre-treatment) started on week 1 for all intervention groups (Groups 3‒7). During week 2, colitis was induced via 3% DSS in all groups except the control and groups 4‒6. DSS was withdrawn in week 3 while interventions continued. On day 21, mice were euthanized. Colons were harvested for measurement, histology, and immunohistochemistry. Colonic fecal contents were collected for 16S rRNA sequencing and metabolomics.

### Hematoxylin and eosin staining and AB-PAS staining

Colonic tissues fixed with 4% paraformaldehyde were thoroughly rinsed under running tap water to remove excess fixative. Tissues were then dehydrated through a graded series of ethanol solutions, cleared in xylene, and embedded in paraffin wax. Serial sections were mounted on glass slides and dried overnight at 37°C for subsequent staining procedures.

For general histopathological evaluation, sections were deparaffinized, rehydrated, and stained with Hematoxylin and eosin (H&E) according to standard protocols. Briefly, sections were stained in Harris’s hematoxylin (G1150; Solarbio, Beijing, China) for 3–5 min, differentiated in 1% acid alcohol, blued in Scott’s tap water, and counterstained with eosin Y (YX19038; YXBIO, Shanghai, China) for 1–2 min.

To assess goblet cells and the mucus layer, adjacent sections were stained with AB-PAS using the commercial kit (RS3790; G-CLONE Biotech) following the manufacturer’s instructions. Briefly, sections were deparaffinized and rehydrated, then stained with Alcian Blue solution for 10‒15 min to visualize acidic mucins. After rinsing, sections were treated with periodic acid solution for 5‒10 min, followed by Schiff’s reagent for 10‒20 min in the dark. Finally, sections were rinsed, counterstained with hematoxylin if required, dehydrated, cleared, and mounted. This method specifically stains acidic mucins blue and neutral mucins magenta.

All stained sections were examined under a BX53 microscope (Olympus, Tokyo, Japan) by two independent pathologists who were blinded to the experimental groups. The severity of colitis was assessed based on a well-established scoring system [21], and microscopic images were captured for documentation.

### Immunohistochemical analysis

To investigate the restorative effects of tanshinones on intestinal barrier integrity, Immunohistochemical (IHC) analysis was performed to assess the expression and localization of tight junction proteins, including ZO-1, occludin, mucin1 and mucin4. Paraffin sections were baked at 60°C for 1 h, deparaffinized in xylene, and rehydrated. Antigen retrieval was performed in citrate buffer (95‒100°C, 15 min), followed by cooling to room temperature. After PBS washes, sections were blocked with rabbit serum at room temperature for 10 min, then incubated overnight at 4°C with primary antibodies (mentioned above). The next day, slides were washed and incubated with secondary antibody (HRP-labeled Goat Anti-Rabbit/Mouse IgG, KIT-5020; Fuzhou Maixin Biotech, Fuzhou, China). For coloration, DBA provided in the enhanced DAB Kit (DAB-2031; Fuzhou Maixin Biotech) was applied for 3 min, followed by hematoxylin counterstaining, dehydration, and mounting. Staining was visualized under a microscope. Positive cell area density was measured in five 200× fields using the BX53 microscope and converted to optical density (OD) with Image-Pro Plus 6.0 (version 6.0.0.260, Media Cybernetics, Rockville, USA) for statistical analysis.

### Quantitative analysis of inflammatory cytokines

To assess the systemic inflammatory response upon tanshinones treatment, the levels of 13 key cytokines (G-CSF, IFN-γ, IL-10, IL-12p70, IL-17, IL-1β, IL-2, IL-23p19, IL-4, IL-6, KC, MCP-1, and TNF-α) in mouse serum were quantified using the commercial multiplex bead-based immunoassay kit (QAM-INF-1; RayBiotech, Peachtree Corners, USA) according to the manufacturer’s instructions. Briefly, 25 μL of diluted serum (diluted 2-fold with PBS), multiplex bead cocktail, and standards were incubated for 2 h at room temperature with shaking. After washing, a biotinylated detection antibody cocktail and subsequently a PE-streptavidin conjugate were added with incubation and wash steps following the protocol. The beads were then resuspended and acquired on a BD FACSCelesta™ flow cytometer (BD Biosciences, Franklin Lakes, USA) equipped with blue and red lasers. Protein concentrations for each cytokine were determined based on their respective standard curves generated from the mean fluorescence intensity in the PE channel.

### Gut microbiota analysis by 16S rRNA sequencing

Given the critical role of gut microbiota in UC pathogenesis, we investigated whether the therapeutic effects of tanshinones are associated with compositional changes in the microbial community. 16S rRNA gene sequencing and subsequent bioinformatics analysis were performed by Shanghai Bioprofile Biotechnology Company (Shanghai, China).

Total genomic DNA was extracted from fecal samples using an Omega Soil DNA kit (D5625-01; Omega Bio-Tek, Norcross, USA). The quality and quantity of the DNA were assessed using a NanoDrop NC2000 spectrophotometer (Thermo Fisher Scientific, Waltham, USA) and confirmed by 1.2% agarose gel electrophoresis. The hypervariable V3–V4 region of the bacterial 16S rRNA gene was amplified using the barcoded universal primers 338F (5′-ACTCCTACGGGAGGCAGCAG-3′) and 806R (5′-GGACTACHVGGGTWTCTAAT-3′). PCR amplification was performed under the following conditions: initial denaturation at 98°C for 5 min; 25 cycles of denaturation at 98°C for 30 s, annealing at 53°C for 30 s, and extension at 72°C for 45 s; followed by a final extension at 72°C for 5 min.

The resulting amplicons were purified using Vazyme VAHTSTM DNA Clean Beads (N411-01; Vazyme, Nanjing, China), quantified with the Quant-iT PicoGreen dsDNA Assay Kit (P7581; Invitrogen, Carlsbad, USA), and pooled in equimolar ratios. The pooled library was subjected to paired-end sequencing (2 × 250 bp) on an Illumina NovaSeq platform (20028401; Illumina, San Diego, USA).

After sequencing, raw reads were processed using QIIME2 (version 2019.4). Quality-filtered reads were clustered into amplicon sequence variants (ASVs) using the DADA2 plugin. Microbial α-diversity (Chao1, Shannon, and Simpson indices) and β-diversity were calculated. Principal coordinate analysis (PCoA) based on Bray-Curtis dissimilarity was performed to visualize microbial community structure. Additional statistical analyses and visualizations were conducted using the vegan, heatmap, and ggplot2 packages in R (version 3.2.0).

### Metabolomics analysis

To further explore the functional output of the gut microbiota and the host metabolic response, untargeted metabolomic profiling was performed on the fecal samples collected alongside those used for microbial analysis. All experimental procedures and subsequent statistical analyses were conducted by Shanghai Bioprofile Biotechnology. Briefly, frozen fecal samples were lyophilized, homogenized using a tungsten bead grinder, and metabolites were extracted with 1 mL of pre-cooled methanol/acetonitrile/water solution (2:2:1, v/v/v). After ultrasonic shaking and incubation at –20°C for 4 h, the extracts were centrifuged at 14,000 *g* for 20 min at 4°C. The resulting supernatant was collected and vacuum-dried for further analysis.

For chromatographic separation, the dried extracts were reconstituted and analyzed using a Shimadzu Nexera X2 LC-30AD UHPLC system (Shimadzu, Kyoto, Japan) coupled with a Q-Exactive Plus mass spectrometer (Thermo Fisher Scientific). Separation was performed on an ACQUITY UPLC® HSS T3 column (2.1 × 100 mm, 1.8 μm; Waters, Milford, USA) maintained at 40°C. The autosampler temperature was set at 4°C, and the injection volume was 4 μL. The mobile phase consisted of solvent A (0.1% formic acid in water) and solvent B (0.1% formic acid in acetonitrile). The gradient elution program at a flow rate of 0.3 mL/min was as follows: 0‒2 min, 0% B; 2‒6 min, linear increase from 0% to 48% B; 6‒10 min, linear increase from 48% to 100% B; 10‒12 min, held at 100% B; 12‒12.1 min, linear decrease from 100% to 0% B; and 12.1‒15 min, held at 0% B for column equilibration.

Mass spectrometric data acquisition was carried out on a Q-Exactive Plus instrument using heated electrospray ionization (HESI) in both positive and negative ion modes. The ion source parameters were set as follows: spray voltage, 3.8 kV (positive) and 3.2 kV (negative); capillary temperature, 320°C; sheath gas, 30 (arbitrary units); auxiliary gas, 5 (arbitrary units); probe heater temperature, 350°C. The mass spectrometer was operated in full-scan data-dependent acquisition (ddMS ^2^) mode. Full-scan MS spectra were acquired over a mass range of m/z 75–1050 with a resolution of 70,000 at m/z 200. For MS/MS acquisition, the top 10 most intense ions from each full scan were selected for fragmentation using higher-energy collisional dissociation (HCD) with a stepped normalized collision energy of 20, 30, and 40. Dynamic exclusion was enabled to avoid repeated sequencing of the same ions.

Raw data were processed using MS-DIAL software for peak alignment, retention time correction, and feature extraction. Metabolite identification was performed by matching accurate mass (mass error < 10 ppm) and MS/MS spectra (mass error < 0.02 Da) against the HMDB, Massbank, and a custom standard database. Features present in more than 50% of samples in at least one group were retained for further analysis.

To visualize the intrinsic clustering of metabolic profiles, we first performed principal component analysis (PCA). Subsequently, partial least squares-discriminant analysis (PLS-DA), orthogonal partial least squares-discriminant analysis (OPLS-DA) and permutation tests were employed to maximize the separation between groups and assess model quality, thus preventing overfitting. Metabolites with a variable importance in projection (VIP) score≥1, fold change ≥ 1.5 or ≤ 0.6, and *P*-value < 0.05 (two-tailed Student’s *t*-test) were considered statistically significant differential metabolites.

### Western blot analysis

The colorectal tissue were lysed with Piere ^TM^ RIPA buffer (89901; Thermo Fisher Scientific) containing a Halt ^TM^ protease and phosphatases inhibitor cocktail (1861281; Thermo Fisher Scientific). The protein concentration was determined using a Pierce ^TM^ BCA kit (23225; Thermo Fisher Scientific). The 20 μg of protein samples were separated by 10% or 12.5% SDS-PAGE and then transferred to PVDF membranes. After blocking with 5% BSA in TBS-T for 1h, membranes were incubated with the primary antibody. The following antibodies were used: anti-IL-1β (1:500), anti-IL-6 (1:500), anti-occludin (1:800), anti-mucin1 (1:800), anti-mucin4 (1:800), and β-actin (1:5000) were used as loading controls. Goat-anti-rabbit IgG (1:5000, 31460; Thermo Fisher Scientific) or goat anti-mouse IgG conjugated to horseradish peroxidase (HRP) (1:5000, 31430; Thermo Fisher Scientific) were used as the secondary antibody. The signals were captured using a ChemiDoc MP imaging system (Bio-Rad Laboratories, Hercules, USA) and analyzed with ImageJ software (National Institutes of Health, Bethesda, USA).

### Statistical analysis

Experimental data are expressed as the mean ± standard deviation (SD). Statistical analyses were performed using GraphPad Prism software and R software (version 3.2.0). For multiple group comparisons, if the data met the assumptions of parametric tests, one-way analysis of variance (ANOVA) was applied, followed by Tukey’s honest significant difference (HSD) post hoc test for all pairwise comparisons. When the assumptions of parametric tests were violated, the non-parametric Kruskal-Wallis test was used, followed by Dunn’s test for post hoc comparisons. Comparisons between two groups were conducted using a two-tailed unpaired Student’s *t*-test (parametric) or the Mann-Whitney U test (non-parametric), as appropriate. A *P*-value of less than 0.05 was considered statistically significant.

## Results

### Administration of tanshinones ameliorates pathological features of DSS-induced colitis

**Figure 2:**
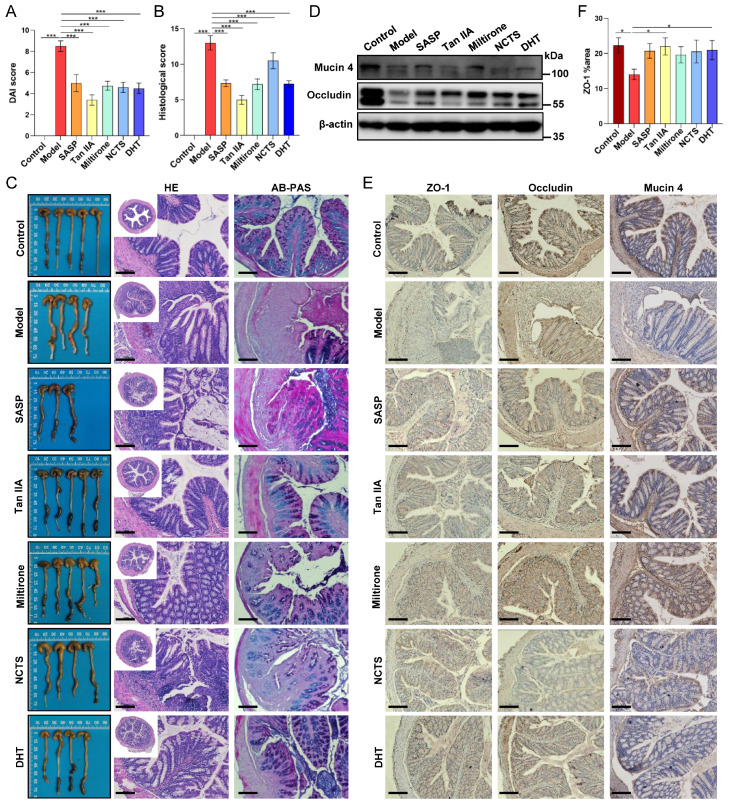
Figure 2 Tanshinones treatment ameliorate pathological features of DSS-induced colitis in mice (A) Disease activity index (DAI) scores. (B) Histological scores based on H&E staining. (C) Representative images of colon length, H&E and AB-PAS staining of colonic tissues. Scale bar: 200 μm. (D) Western blot analysis was conducted to quantify the protein levels of occludin and mucin4 across experimental groups. (E) Representative images of immunohistochemical staining for tight junction proteins ZO-1 and occludin. Scale bar: 200 μm. (F) Quantitative analysis of the positive staining area for ZO-1. Data are presented as the mean ± SD from at least three independent experiments (n = 4‒5). Statistical analysis was performed by one-way ANOVA with Tukey’s post-hoc test. *P < 0.05 and ***P < 0.001 vs Model group.

The therapeutic effects of various *Salvia miltiorrhiza* tanshinones were evaluated in a murine model of DSS-induced colitis. SASP, a widely used prodrug clinically effective in treating mild-to-moderate UC by enhancing fecal butyrate production [22], served as the positive control. Compared to the Control group, mice in the Model group exhibited progressive weight loss ( Supplementary Figure S1A) and a significantly increased DAI score ( Figure 2A), accompanied by colon shortening ( Supplementary Figure S1B). Treatment with SASP and the four tanshinones (Tan IIA, miltirone, NCTS, and DHT) significantly improved the DAI scores compared to the Model group.

Histological analysis of colon tissues by H&E staining revealed severe damage in the Model group, including mucosal erosion, crypt loss, and inflammatory infiltration, resulting in a high histological score. Inversely, all treatment groups, showed significant alleviation of these pathological changes and reduced histological scores ( Figure 2B,C). Examination of major organs by H&E staining revealed no obvious pathological alterations in any group compared to the Control group ( Supplementary Figure S1C).

The colonic mucus barrier, secreted by goblet cells, constitutes the first line of host defense protecting the intestinal epithelium from invasion [23]. The integrity of the colonic mucus barrier, assessed by AB-PAS staining, was severely compromised in the Model group, as evidenced by goblet cell depletion and reduced mucus coverage. This defect was significantly reversed by treatment with SASP, Tan IIA, miltirone, and DHT, which increased goblet cell numbers and restored mucus layer continuity, with the effect being most notable in the Tan IIA group ( Figure 2C).

Epithelial barrier defect is a hallmark pathological feature of UC [24]. We assessed whether tanshinones treatment restores barrier function through western blot analysis of occludin and mucin4 protein levels ( Figure 2D) and immunohistochemical staining of ZO-1, occludin, and mucin4 ( Figure 2E,F and Supplementary Figure S1D). Consistent with the morphological findings, tanshinones reversed the impaired expression of mucosal barrier proteins (ZO-1, occludin, and mucin4) in the UC model, collectively suggesting a reinforcement of the epithelial barrier.

### Tanshinones suppress the activation of pro-inflammatory cytokines

**Figure 3:**
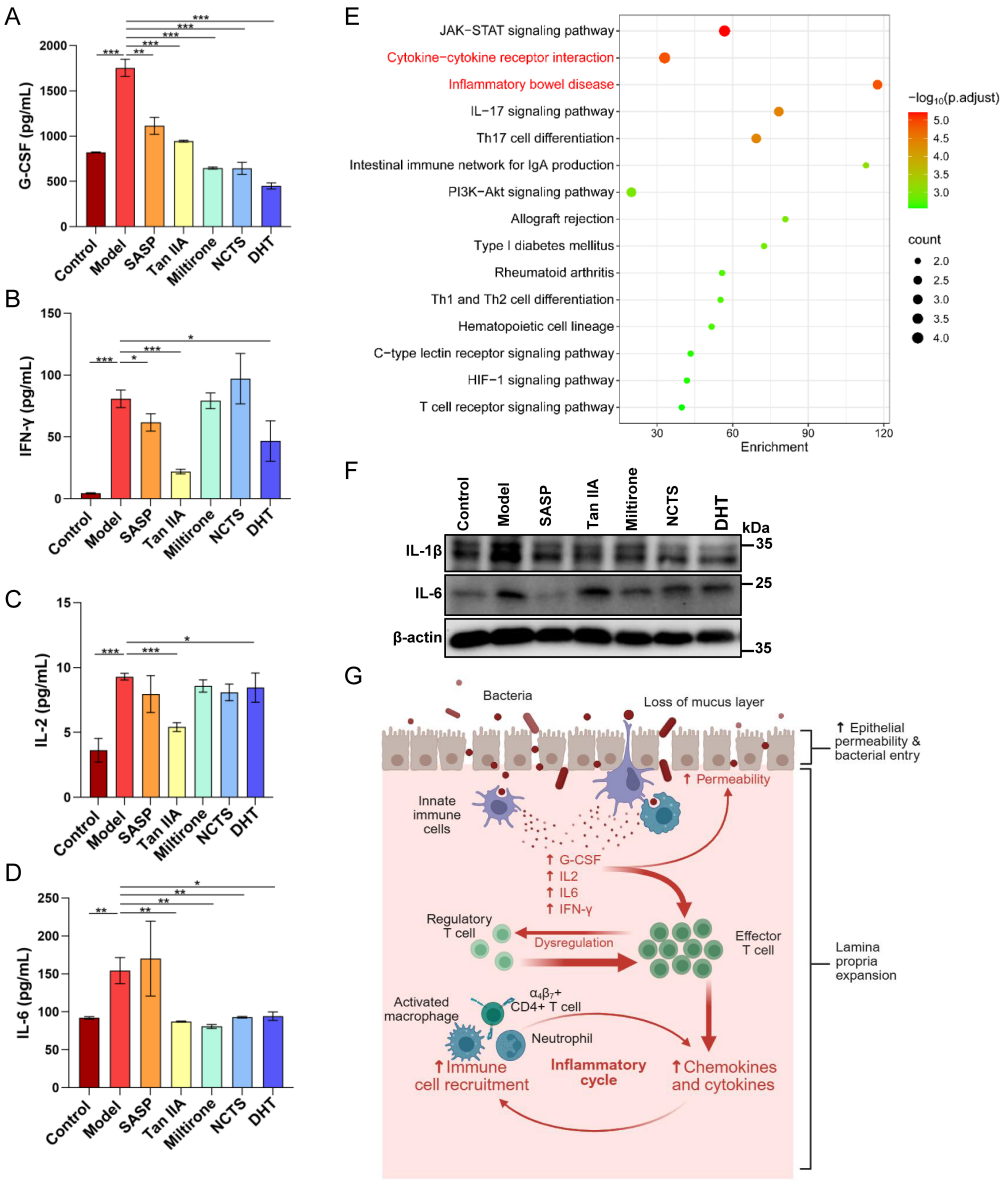
Figure 3 Tanshinones alleviate DSS-induced colitis by modulating immune-inflammatory pathways (A‒D) Serum levels of inflammatory cytokines: (A) G-CSF, (B) IFN-γ, (C) IL-2, and (D) IL-6. Data are presented as the mean ± SD, *P < 0.05, **P < 0.01, ***P < 0.001. (E) KEGG pathway enrichment analysis showing immune-related pathways that were predicted to be significantly altered by tanshinones treatment. Bubble size indicates the number of enriched proteins; color intensity represents the statistical significance, –log10(P-adjust). (F) Western blot analysis was conducted to detect the protein levels of IL-1β and IL-6. (G) Schematic summary illustrating the proposed mechanism: impairment of intestinal barrier integrity facilitates bacterial translocation, which subsequently activates pro-inflammatory macrophages and effector T cells. This activation triggers the release of pro-inflammatory cytokines (IL-6 and IFN-γ, etc.), and initiates a cycle of chronic inflammation. Data are presented as the mean ± SD (n = 5 mice per group). Statistical significance was determined by one-way ANOVA with Tukey’s post-hoc test.

Inflammation is a hallmark of colitis, and the levels of inflammatory cytokines are closely associated with disease severity [25]. To assess the anti-inflammatory effects of tanshinones, serum levels of multiple cytokines were quantified. DSS-induced colitis significantly elevated the levels of pro-inflammatory cytokines, including G-CSF, IFN-γ, IL-2, IL-6, IL-1β, IL-4, and IL-17 compared to the Control group **(** Figure 3A‒D and Supplementary Figure S2A–C), while the level of TNF-α remained unchanged ( Supplementary Figure S2D). Treatment with SASP significantly reduced G-CSF and IFN-γ levels. All four tanshinone components effectively suppressed key pro-inflammatory cytokines, with Tan IIA, miltirone, NCTS, and DHT all markedly lowering G-CSF and IL-6. Furthermore, Tan IIA specifically decreased IFN-γ and IL-2, while DHT significantly reduced IFN-γ ( Figure 3B,C).

Gene ontology (GO) and KEGG pathway analyses were performed to elucidate the underlying mechanisms. The results showed that tanshinones treatment were significantly enriched in biological processes related to T cell activation, B cell response, and JAK-STAT signaling ( Supplementary Figure S2E). KEGG analysis suggested the potential involvement of key pathways, including the JAK-STAT, IL-17 signaling pathway, and cytokine-cytokine receptor interaction ( Figure 3E). To Further determine the local anti-inflammatory activity of tanshinones, we measured the protein levels of IL-1β and IL-6 in colonic tissues via western blot analysis. Consistent with a marked inflammatory response in the UC model group, both IL-1β and IL-6 expression levels were significantly elevated compared to controls ( Figure 3F). Administration of each of the four tanshinones substantially reduced the expression of these cytokines, albeit to varying degrees. These findings demonstrate that tanshinones exert direct anti-inflammatory effects in UC.

Based on these findings, we propose a mechanistic hypothesis: Impairment of intestinal barrier integrity facilitates bacterial translocation, which subsequently activates pro-inflammatory macrophages and effector T cells. This activation triggers the release of cytokines, such as IL-6 and IFN-γ, and initiates a cycle of chronic inflammation ( Figure 3G).

### Tanshinones restore gut microbial homeostasis in DSS-induced colitis

**Figure 4:**
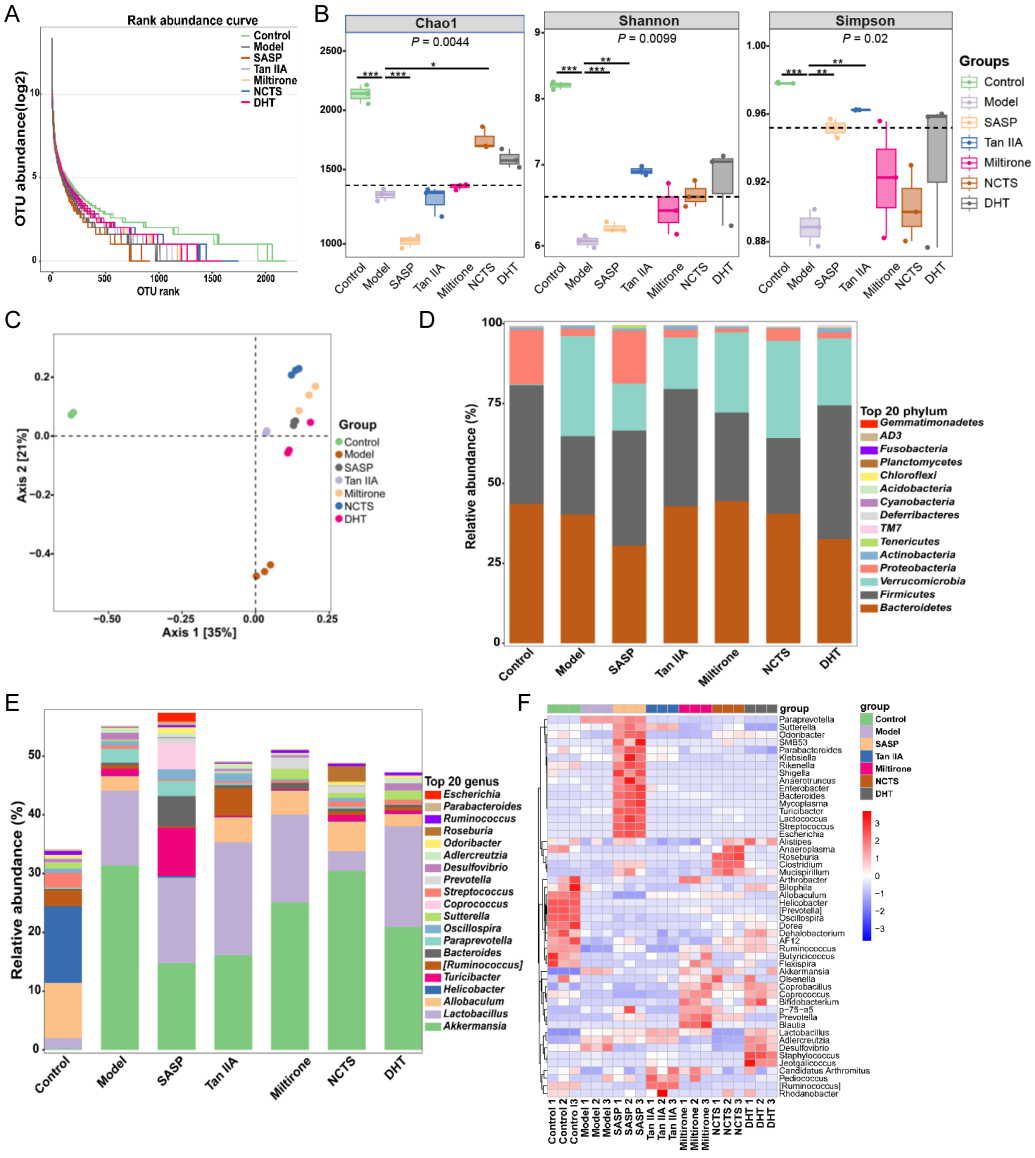
Figure 4 Tanshinones reshape the gut microbiota in DSS-induced colitis (A) Rank-abundance curves, where the extent of the curve indicates richness and the slope reflects evenness. (B) Box plots of alpha diversity indices (Chao1, Shannon, and Simpson). Values are presented as the mean ± SD (n = 3 per group). Significant differences were determined by one-way ANOVA followed by Tukey’s post-hoc test (*P < 0.05, **P < 0.01, ***P < 0.001). (C) Principal coordinates analysis (PCoA) based on Bray-Curtis dissimilarity, revealing distinct clustering of microbial communities according to treatment. (D,E) Stacked bar plots showing the relative abundance of the most abundant bacterial (D) phyla and (E) genera. Only the top 15–20 most abundant taxa are shown. (F) Heatmap depicting the Z-score normalized relative abundance of genera that exhibited significant differences (P < 0.05) across groups. Rows and columns were clustered using hierarchical clustering.

To determine whether tanshinones alleviate colitis by modulating the gut microbiota, we performed 16S rRNA sequencing on colonic contents. We first assessed the overall structural changes in the gut microbiota. Species accumulation curves confirmed adequate sequencing depth for capturing microbial diversity ( Supplementary Figure S3A). Rank-abundance curves showed that the Model group exhibited reduced community evenness, as evidenced by a steeper curve, which was ameliorated by tanshinones treatment ( Figure 4A). This was further supported by the Venn diagram, which illustrated a loss of unique operational taxonomic units (OTUs) in the Model group ( Supplementary Figure S3B). Consistently, alpha diversity indices (Chao1, Shannon, and Simpson) revealed a significant decline in microbial richness and evenness in the Model group, both of which were restored by tanshinones treatment ( Figure 4B and Supplementary Table S1). PCoA based on Bray-Curtis dissimilarity showed clear separation between the Model group and other groups ( Figure 4C), further supported by weighted UniFrac distance ( Supplementary Figure S3C) and PCA ( Supplementary Figure S3D), indicating distinct microbial community structures.

To identify which specific bacterial taxa are responsible for these changes, we profiled the community composition. At the phylum level, the Model group showed an increase in *Verrucomicrobia* and a decrease in *Proteobacteria* and *Firmicutes*, forming a dysbiotic state typical of intestinal inflammation, which were reversed by Tan IIA treatment ( Figure 4D). Genus-level analysis revealed that Tan IIA suppressed pathogens such as *Akkermansia*, *Desulfovibrio*, and *Adlercreutzia*, while promoting beneficial genera including *Allobaculum* and *Oscillospira* ( Figure 4E,F and Supplementary Table S2).

**Figure 5:**
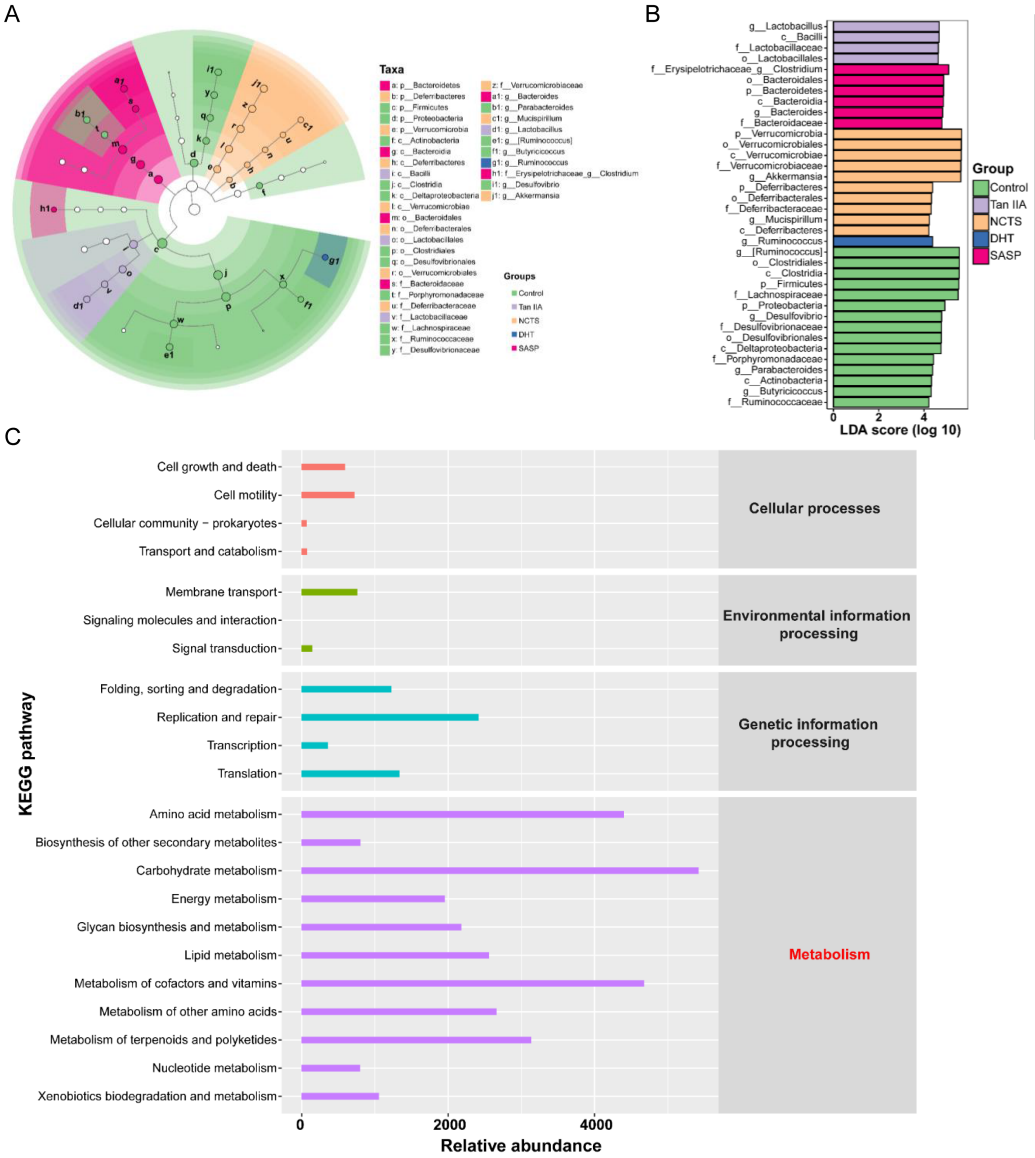
Figure 5 Tanshinones modulate gut microbiota composition and predicted function (A) Cladogram generated by LEfSe analysis, illustrating the phylogenetic distribution of bacterial taxa that were significantly enriched in specific groups. The color of the circles indicates the group in which the taxon is enriched. (B) Histogram of the linear discriminant analysis (LDA) scores from LEfSe analysis. The length of the bar represents the effect size of differentially abundant taxa (LDA score > 4.0) that discriminate each group from the others. (C) Bar plot of the inferred relative abundance of KEGG pathways (Level 2) based on PICRUSt2 prediction. Displayed pathways showed significant differences (P < 0.05, ANOVA) among groups.

To robustly identify the most differentially abundant taxa acting as biomarkers, we performed linear discriminant analysis (LDA) effect size (LEfSe) analysis. While the Model group did not exhibit strongly discriminatory features against the Control in this analysis, each treatment group presented a unique set of enriched microbial biomarkers ( Figure 5A,B). LEfSe analysis identified *Lactobacillus* as a key biomarker enriched in the Tan IIA group (LDA > 4), both are short-chain fatty acid producers and are frequently associated with gut health and anti-inflammatory effects. In contrast, SASP was primarily associated with an increase in *Bacteroides* ( Figure 5A,B).

We next sought to understand the functional implications of these structural shifts. Prediction of metagenomic functions using PICRUSt2 revealed profound alterations at the pathway level. Comparison between the Control and Model groups uncovered the core functional deficits in colitis: key metabolic pathways including primary and secondary bile acid biosynthesis, linoleic acid metabolism, and amino acid metabolism were significantly downregulated, whereas pathogenicity-associated pathways such as bacterial invasion of epithelial cells and beta-lactam resistance were markedly enriched ( Figure 5C and Supplementary Figure S3E). Crucially, tanshinones treatment effectively reversed this dysfunctional profile. The predicted functional profile suggested that Tan IIA administration may be linked to the upregulation of critical metabolic pathways like bile acid biosynthesis and D-arginine metabolism, and a concurrent downregulation of detrimental pathways including bacterial invasion of epithelial cells and pathogenic *E*. *coli* infection. A similar restorative trend was observed in the miltirone group. The positive control SASP also suppressed pathogenic pathways but showed a distinct functional modulation pattern compared to tanshinones. Co-occurrence network analysis further indicated that Tan IIA promoted a more complex and stable microbial ecology resembling that of the healthy state ( Supplementary Figure S4A‒C and Supplementary Table S3).

Collectively, these findings indicate that the therapeutic effect of tanshinones is intrinsically linked to a functional reprogramming of the gut microbiota, rectifying the metabolic deficiencies and suppressing the pro-inflammatory potential instigated by DSS.

### Tanshinones remodel the gut metabolome and reverse DSS-induced metabolic disruptions

**Figure 6:**
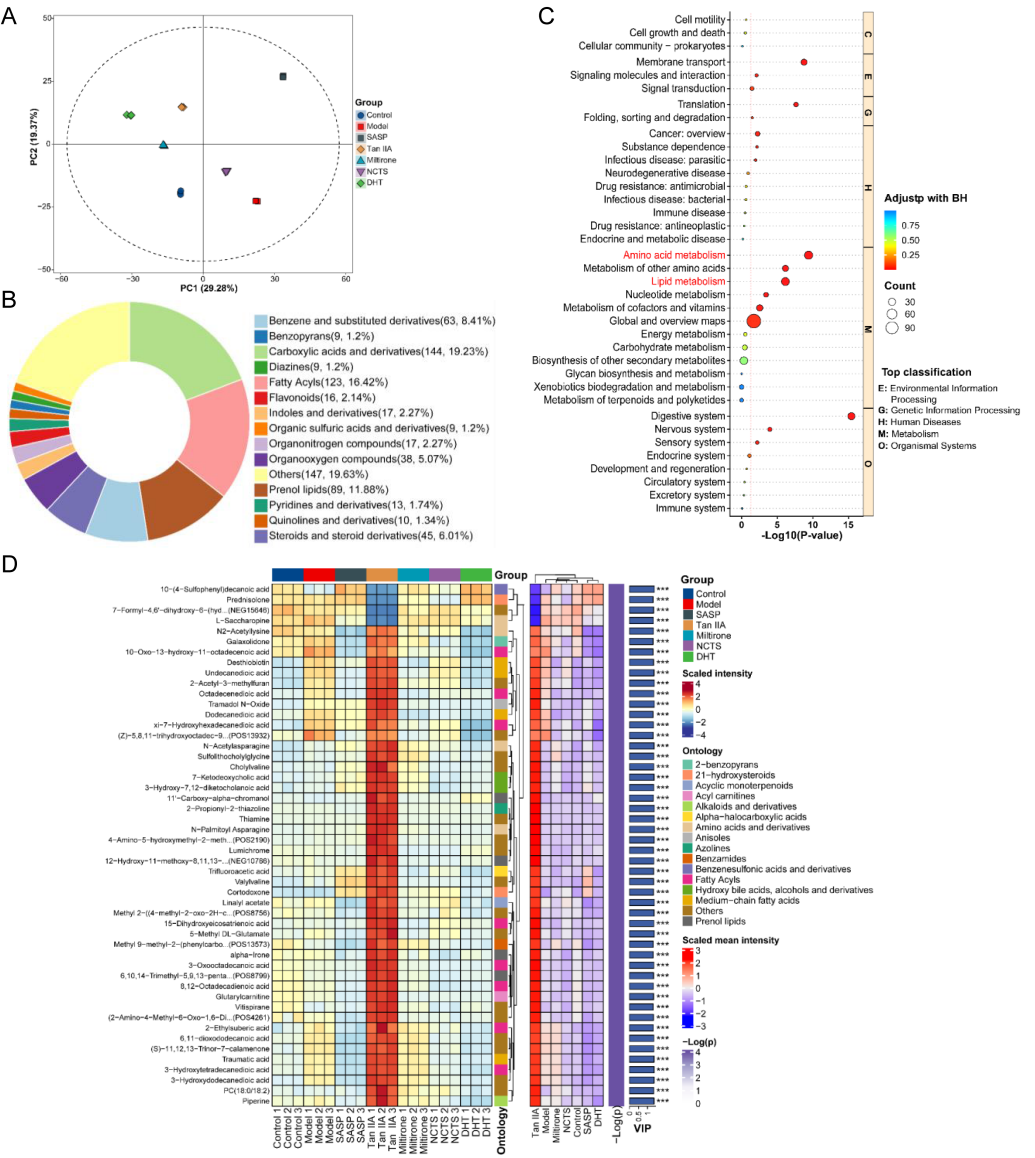
Figure 6 Tanshinones induce systemic metabolic reprogramming in a UC model (A) PCA score plot of the fecal metabolome, demonstrating clear separation among groups. (B) Bar chart showing the distribution of identified metabolites by their chemical superclass. (C) Enriched KEGG pathways based on differential metabolites. The size of the bubble is proportional to the number of metabolites mapped to the pathway. The color scale indicates the level of statistical significance (–log10 of the Benjamini-Hochberg adjusted P-value). The X-axis (Enrichment Factor) represents the degree of enrichment. (D) Unsupervised hierarchical clustering heatmap of metabolites that were significantly altered (VIP > 1.0 and P < 0.05) across groups. Metabolite abundances were transformed to Z-scores (row-wise). Red and blue colors denote levels above and below the mean abundance, respectively.

Previous studies have demonstrated that gut microbiota-derived metabolites play an essential role in maintaining intestinal mucosal homeostasis [ 25, 26] . Having established that tanshinones restore gut microbial homeostasis, we next wondered whether these structural changes translate into functional metabolic alterations, therefore, we performed untargeted metabolomic profiling on fecal samples. PCA, PLS-DA, and OPLS-DA revealed a clear separation among the Control, Model, and tanshinone-treated groups ( Figure 6A and Supplementary Figure S5A,B), indicating distinct metabolic profiles. We identified 1605 metabolites in total ( Supplementary Table S4), and 770 were defined as differentially abundant metabolites (DAMs) based on a variable importance in projection (VIP) score > 1.0 and a *P*-value < 0.05 ( Supplementary Figure S5C,D and Supplementary Table S5). Classification of the detected metabolites showed that carboxylic acids and derivatives (19.23%) and fatty acyls (16.42%) constituted the largest proportions of the metabolome, followed by prenol lipids (11.88%) and benzene/substituted derivatives (8.41%) ( Figure 6B).

KEGG pathway enrichment analysis of the DAMs suggested that DSS induction caused widespread metabolic perturbations. Compared to the Control group, the Model group exhibited significant enrichment in pathways related to amino acid metabolism, arginine metabolism, and lipid metabolism (linoleic acid metabolism, *etc*.) ( Figure 6C and Supplementary Figure S5E). Notably, tanshinones treatment effectively reversed these alterations. Furthermore, pathways associated with infectious diseases and immune diseases were also significantly modulated, aligning with the pathophysiology of UC.

Hierarchical clustering analysis of the DAMs confirmed a clear segregation between the Model group and the treatment groups ( Figure 6D). We further identified that the reversal of metabolic dysregulation by tanshinones was driven by specific key metabolites. For instance, tanshinones treatment were associated with a marked amplification in the levels of specific bile acid derivatives ( *e*. *g*., 7-ketodeoxycholic acid, sulfolithocholylglycine) and medium-chain fatty acids, which may represent an enhanced host-microbiota metabolic response. This shift occurred alongside the restoration of metabolites linked to amino acid metabolism ( *e*. *g*., reduced L-saccharopine), energy homeostasis ( *e*. *g*., decreased propionylcarnitine), and oxidative stress ( *e*. *g*., normalized 2-hydroxybutyric acid).

Collectively, these results prompt that tanshinones induce a comprehensive reprogramming of the gut metabolome, primarily by rectifying abnormalities in bile acid metabolism and energy metabolism, thereby contributing to the restoration of host metabolic homeostasis in UC.

### Integrated analysis reveals a microbiota-metabolite axis underlying the therapeutic effect of tanshinones

**Figure 7:**
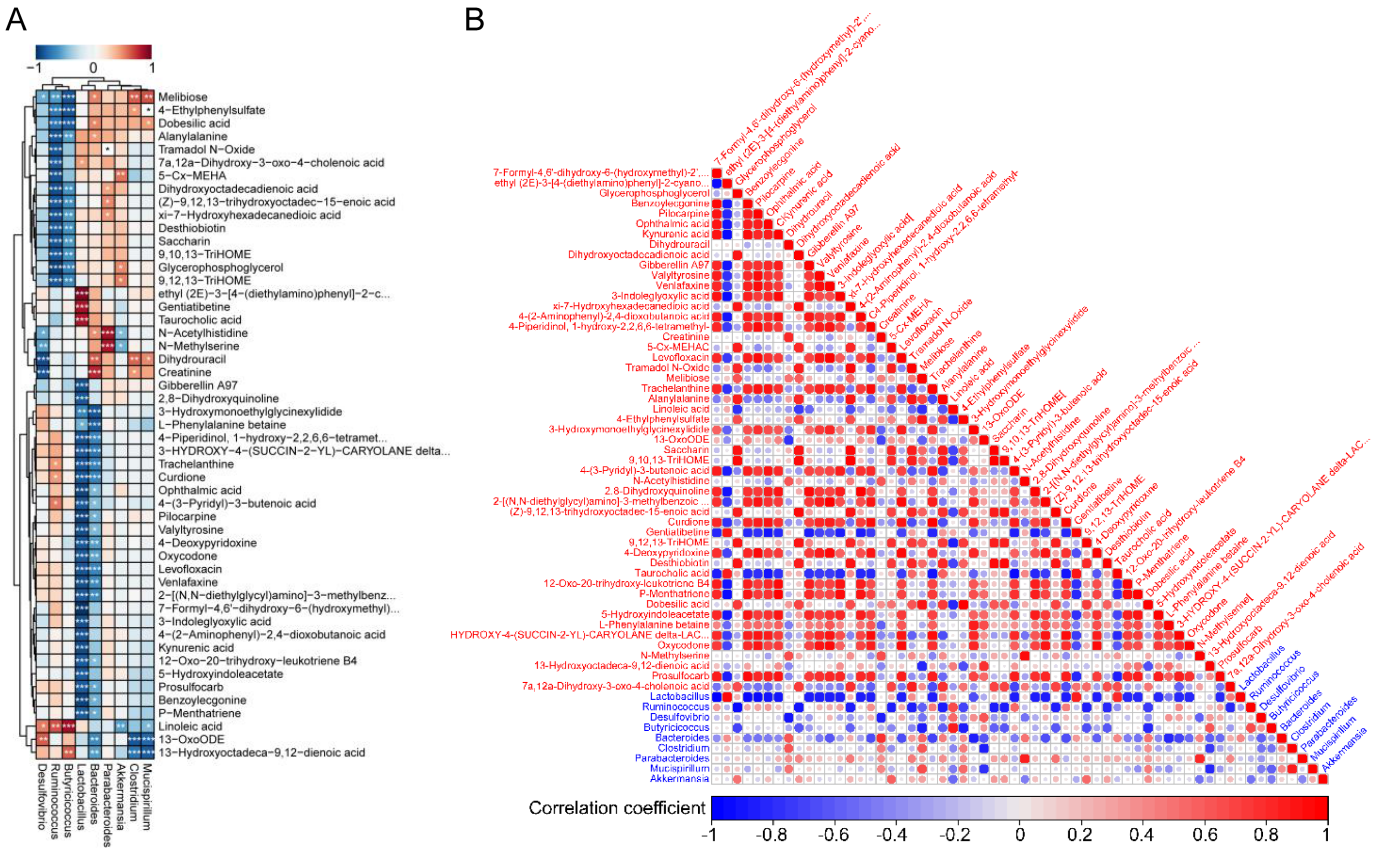
Figure 7 Integrated analysis identifies key modules in the microbiota-metabolite axis of colitis (A) Hierarchical clustering heatmap of Spearman correlation coefficients between differential microbial genera and metabolites. Red and blue colors indicate positive and negative correlations, respectively. The ellipse size and asterisks denote the statistical significance of each correlation (*P < 0.05, **P < 0.01, ***P < 0.001). The clustering reveals distinct co-expression modules. (B) Network visualization of significant microbe-metabolite correlations. Nodes represent microbial genera (blue squares) and metabolites (red circles). The size of a node corresponds to its degree (number of connections), highlighting hub entities. The color intensity of the edges (lines) and metabolite nodes reflects the absolute value of the correlation coefficient (|r|). Only correlations with a significance of P < 0.05 are shown.

To functionally integrate the microbial and metabolic alterations induced by tanshinones, we performed a Spearman correlation network analysis between significantly altered bacterial genera (LDA > 4) and key metabolites. The resulting clustered heatmap ( Figure 7A) revealed extensive correlations, organizing into distinct, co-varying functional modules that link specific microbial taxa to host metabolic outputs. Network visualization ( Figure 7B) further delineated this complex interplay, identifying central microbial hubs and their associated metabolic landscapes.

The analysis delineated two principal, health-associated modules. First, mucin-associated and symbiotic bacteria, notably *Akkermansia* and *Parabacteroides*, emerged as highly connected hubs positively correlated with a broad spectrum of metabolites indicative of mucosal health and anti-inflammatory states, including specific oxylipins and dihydroxyoctadecadienoic acid. Second, a tightly interconnected module was formed by butyrate-producing genera such as *Butyricicoccus* and *Ruminococcus*, whose enrichment was strongly linked to elevated levels of these same inflammation-resolving lipid mediators ( *e*. *g*., 9,12,13-TriHOME). This convergence underscores a cooperative microbial-metabolic axis promoting resolution. In stark contrast, the sulfate-reducing pathobiont Desulfovibrio anchored a separate, disease-associated cluster, characterized by strong positive correlations with pro-inflammatory and cytotoxic metabolites like taurocholic acid and 13-OxoODE. This detrimental cluster was largely dissociated from the beneficial metabolite networks, highlighting a pathogenic metabolic niche.

Collectively, these data demonstrate that tanshinones alleviate UC not merely by modulating microbial composition, but by orchestrating a systems-level reprogramming of host-microbiota co-metabolism. The treatment restructures the ecological network by reinforcing cooperative functional modules, linking mucin-foraging and butyrogenic bacteria to the production of inflammation-resolving lipids, while simultaneously decoupling pathobionts from disrupted bile acid metabolism. This coordinated shift in the functional output of the gut ecosystem underpins the therapeutic efficacy of tanshinones.

### Combined Tan IIA and *M*. *Akk* treatment attenuates colitis through host-microbiota co-metabolic reprogramming

**Figure 8:**
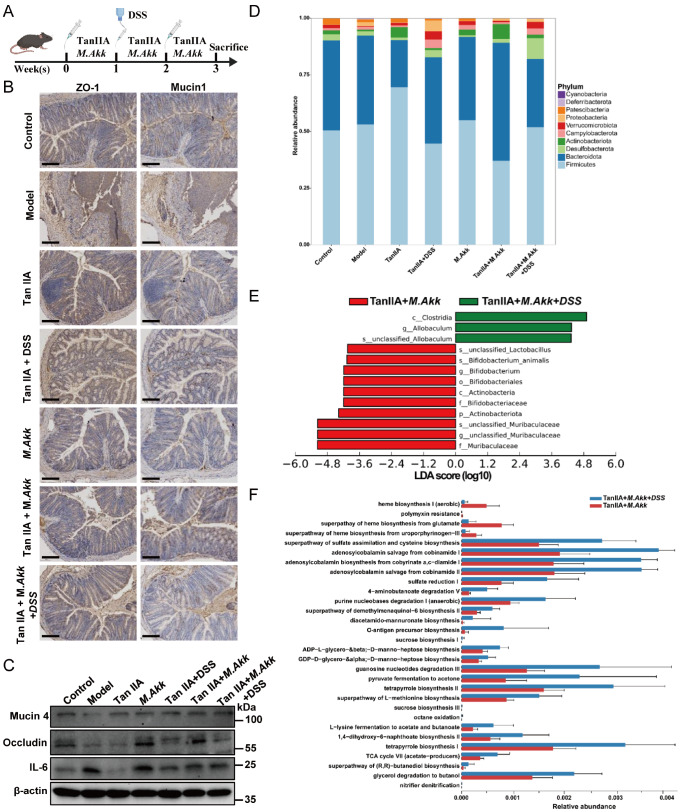
Figure 8 Synergism of Tan IIA and *M*. *Akk* reprograms microbial metabolism and reinforces the intestinal barrier to alleviate colitis (A) Schematic timeline of the experimental design. (B) Histochemical staining demonstrates the restoration of mucosal barrier-related proteins following interventions. (C) Western blot analysis confirms the upregulation of mucosal proteins (mucin4 and occludin) and downregulation of the pro-inflammatory cytokine IL-6. (D) 16S rRNA sequencing reveals the restoration of DSS-induced dysbiosis at the phylum level after treatments. (E) LEfSe analysis identifies distinct microbiota signatures enriched by Tan IIA and M.Akk co-treatment in both healthy and colitis conditions (LDA score > 4). (F) Functional prediction analysis (PICRUSt2) using the MetaCyc database highlights key microbial metabolic pathways modulated by the combined treatment.

To further elucidate the core mechanism by which tanshinones exert their therapeutic effects via microbiota modulation, we investigated the cooperative action of Tan IIA and *M*. *Akk* in UC treatment. First, *in vitro* experiments confirmed that four tanshinones (including Tan IIA) inhibited the growth of *M*. *Akk* in a dose-dependent manner (at concentrations as low as 12 μM and 25 μM) ( Supplementary Figure S6A,B). To investigate the roles of Tan IIA and *M*. *Akk* in the treatment of UC, we administered Tan IIA and/or *M*. *Akk* to both healthy and UC mice ( Figure 8A). The results showed that supplementation with Tan IIA alone, *M*. *Akk* alone, or their combination effectively restored colon length in UC mice ( Supplementary Figure S7A). Histopathological examination further revealed that Tan IIA and *M*. *Akk* reversed inflammatory infiltration and epithelial damage. Specifically, compared with the model group, key mucosal proteins, including mucins (mucin1, mucin4) and tight junction proteins (ZO-1, Occludin), were significantly upregulated, while IL-6 levels were markedly diminished ( Figure 8B,C and Supplementary Figure S7B–G).

The 16S rRNA analysis revealed that at the phylum level, DSS-induced colitis led to a characteristic dysbiosis, marked by a significant reduction in *Firmicutes* (a major SCFA-producing phylum) and a notable expansion of *Proteobacteria* (often containing pro-inflammatory taxa). All treatment regimens-Tan IIA, *M*. *Akk*, and their combination, partially restored this imbalance, with the Tan IIA + *M*. *Akk*+ DSS group showing the strongest recovery of *Firmicutes* and suppression of *Proteobacteria*, alongside a restored *Firmicutes*/ *Bacteroidota* ratio ( Figure 8D). Genus-level analysis further revealed that beneficial butyrate-producing genera such as *Lachnospiraceae* were depleted in the model group, while potentially inflammatory genera including *Escherichia-Shigella* and *Allobaculum* were enriched. Treatment with Tan IIA and/or *M*. *Akk* consistently reversed these changes, promoting the recovery of beneficial taxa and suppressing conditionally pathogenic genera, with the most pronounced effects observed in the combination group ( Supplementary Figure S7H).

LEfSe analysis comparing the Tan IIA + *M*. *Akk* group with the Tan IIA + *M*. *Akk *+ DSS group revealed context-dependent shifts in microbiota composition. Under healthy (non-colitis) conditions, the combined treatment significantly enriched taxa such as *Clostridia* and *Allobaculum* (LDA > 4.0), indicating their strong association with the treatment in the absence of inflammation ( Figure 8E). In the context of DSS-induced colitis, however, the treatment group exhibited a distinct enrichment of recognized probiotic genera, including several *Bifidobacterium* species and an *unclassified Lactobacillus* species, suggesting the Tan IIA + *M*. *Akk* combination fosters a microbiota rich in immunomodulatory and potentially beneficial taxa during inflammation. Functional prediction analyses further elucidated the metabolic implications of these compositional shifts. KEGG pathway analysis revealed that the Tan IIA + *M*. *Akk* treatment in colitis mice specifically enriched microbial pathways related to energy/carbohydrate metabolism and amino acid metabolism, while pathways governing core cellular processes ( *e*. *g*., cell growth and motility) remained unchanged. This indicates a targeted reprogramming of microbial metabolic function rather than a broad disruption of cellular activity ( Supplementary Figure S7I). MetaCyc database analysis provided deeper metabolic resolution, showing significant enrichment in pathways related to polyphenol metabolism and steroid hormone biosynthesis ( *e*. *g*., adrenocorticoid pathways). These findings imply that the TanIIA + *M*. *Akk* intervention steers the gut microbiota towards metabolic processes capable of generating anti-inflammatory compounds (from polyphenols) and modulating host immune responses (via steroid metabolism) ( Figure 8F), collectively supporting a mechanism of action centered on microbial metabolic remodeling.

**Figure 9:**
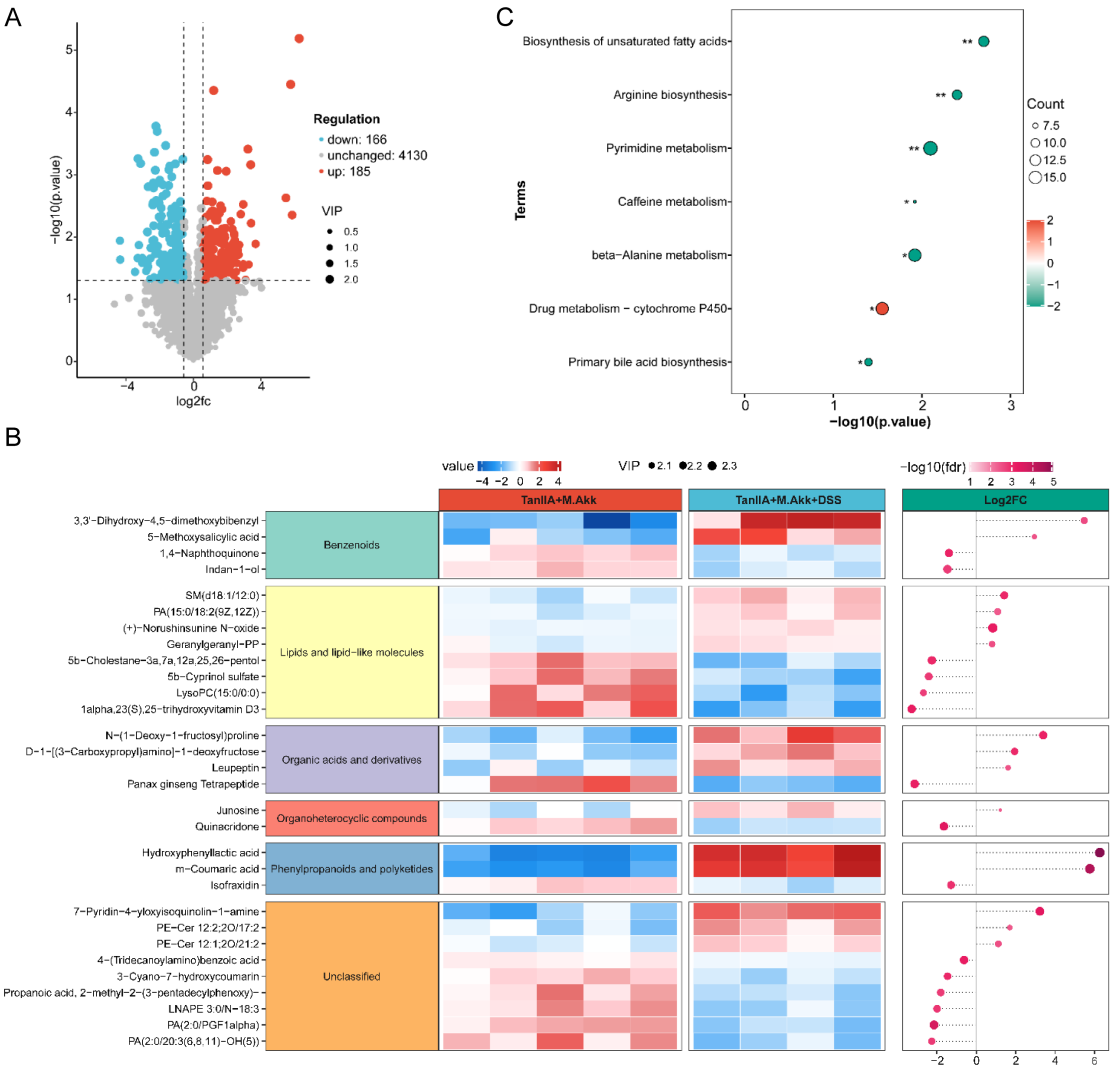
Figure 9 Metabolic alterations induced by DSS challenge in the Tan IIA and *M*. *Akk* co-treatment group (A) Volcano plot displaying differential metabolites (166 downregulated, 165 upregulated; VIP > 1.0, P < 0.05) in the Tann IIA + M.Akk + DSS versus Tan IIA + M.Akk comparison. (B) Heatmap of the top 30 differentially abundant metabolites ranked by VIP score. Rows represent metabolites, columns represent biological replicates per group. Metabolites are annotated by their super-class (right sidebar). Color intensity indicates Z-score-scaled relative abundance. (C) Metabolite set enrichment analysis (MSEA) of the same comparison. Pathways are ranked by enrichment significance, –log10(P-value).

To delineate the functional metabolic outcomes of Tan IIA and *M*. *Akk* intervention, we performed fecal metabolomic profiling across healthy and colitis conditions ( Supplementary Figure S8A). Compared to healthy controls, DSS-induced colitis triggered extensive metabolic dysregulation, with 331 metabolites significantly altered (166 downregulated, 165 upregulated) in the model group ( Figure 9A). Unsupervised clustering of the top 30 differential metabolites clearly segregated the experimental groups and highlighted treatment-responsive compounds ( Figure 9B). Pathway analysis revealed that the therapeutic effect of Tan IIA + *M*. *Akk* was associated with a targeted reprogramming of host-microbial co-metabolism. KEGG enrichment showed significant reversal of colitis-induced perturbations in amino acid metabolism ( *e*. *g*., phenylalanine and tryptophan) and steroid hormone biosynthesis ( Supplementary Figure S8B,C). Equally noteworthy was the enrichment in ‘Metabolism of xenobiotics by cytochrome P450’, indicating enhanced detoxification capacity. Broader metabolite set enrichment analysis (MSEA) further confirmed these shifts and identified concurrent remodeling of lipid homeostasis (biosynthesis of unsaturated fatty acids, primary bile acid biosynthesis) and xenobiotic metabolism ( Figure 9C).

Collectively, our results demonstrate that the combined intervention of Tan IIA and *M*. *Akk* exerts a potent therapeutic effect against colitis through a synergistic, two-pronged mechanism. Structurally, it restores gut microbial homeostasis by enriching beneficial genera ( *e*. *g*., *Bifidobacterium* and *Lactobacillus*) and suppressing pro-inflammatory taxa. Functionally, this restructured microbiota drives a comprehensive reprogramming of the host-microbial metabolic network, rectifying key deficits in amino acid utilization, steroid/bile acid balance, and xenobiotic detoxification. This coordinated remodeling of the microbial ecosystem and its metabolic output underpins the restoration of intestinal barrier integrity, resolution of inflammation, and systemic homeostasis.

## Discussion

Our study identifies functional reprogramming of the gut microbiota-metabolite axis as a core mechanism through which *Salvia miltiorrhiza* lipophilic components, notably tanshinone IIA, ameliorate ulcerative colitis. Integrated multi-omics analysis reveals that the therapeutic effect orchestrates both structural microbial changes ( *e*. *g*., restoration of diversity, suppression of *Turicibacter*, enrichment of *Lactobacillus* and *Coprococcus*) and, crucially, a normalization of host-microbiota co-metabolism. Specifically, treatment with tanshinones rectifies disruptions in key pathways, including the metabolism of anti-inflammatory fatty acid derivatives and bile acids.

The restorative impact of tanshinones on microbial ecology is a cornerstone of its mechanism. Consistent with established signatures of gut dysbiosis in UC [ 27– 29] , our model exhibited a loss of beneficial phyla like *Firmicutes* and an expansion of *Verrucomicrobia*. Tanshinone IIA effectively reversed these trends. More importantly, at the genus level, salutary bacteria, such as *Lactobacillus* and *Coprococcus*, both known for their roles in immune regulation and SCFA production, were enriched by tanshinone treatment [ 30, 31] . Concurrently, tanshinones suppressed pro-inflammatory pathobionts like *Turicibacter*, which is associated with chronic inflammation and even carcinogenic metabolite production [ 32, 33] . This coordinated shift in community structure underscores tanshinones’ capability to reestablish a homeostatic microbial environment.

Parallel to these structural changes, tanshinones induced a profound metabolic reprogramming. Metabolomic analysis revealed a reversal of DSS-induced perturbations in pivotal pathways, including linoleic acid and primary bile acid metabolism—alterations that are hallmarks of the UC metabolome [ 34, 35] . Notably, we observed a marked increase in *Akkermansia* abundance in the DSS model group, which is interpreted as an opportunistic overgrowth fueled by extensive mucus layer degradation during acute colitis. While generally beneficial in homeostasis, its uncontrolled proliferation in a damaged gut may exacerbate barrier dysfunction. Tanshinones treatment reversed this overgrowth, likely by repairing the mucosal niche.

The overall therapeutic effect appears to be driven by the reinstatement of cooperative functional modules within the gut ecosystem, rather than the action of isolated bacterial species. For instance, the predicted enrichment of pathways related to bile acid metabolism aligns with the recovery of colonic mucosal integrity, as certain microbial bile acid transformations are known to regulate host innate immunity and barrier function [ 36, 37] . Correlation network analysis further supports this modular view, revealing clusters where microbial taxa are linked to suites of beneficial metabolites. Intriguingly, in the post-treatment state, correlation network analysis identified *Akkermansia* as a central hub, showing a strong association with anti-inflammatory lipids such as dihydroxyoctadecadienoic acid, a linoleic acid derivative. This suggests that beyond its role in mucin degradation, *Akkermansia* may contribute to intestinal health through immunomodulatory lipid metabolism following tanshinones intervention, indicating a shift in its ecological role from a potential pathobiont in active inflammation toward a beneficial commensal during recovery [ 38, 39] .

Integration of microbiome and metabolome data provided functional insights into the observed correlations. For instance, the strong positive correlation between *Desulfovibrio* and taurocholic acid suggests that tanshinones may alleviate colitis partly by suppressing sulfate-reducing bacteria and thereby reducing the pool of this primary bile acid, which can be inflammatory [40]. Furthermore, the link between the butyrate-producer *Butyricococcus* and oxylipins ( *e*. *g*., 9,10,13-TrHOME) posits an intriguing mechanism: the benefits of butyrate may be amplified through oxylipin-mediated modulation of barrier function and immune responses [ 41, 42] .

Our multi-omics study elucidates that the anti-colitis effects of *Salvia miltiorrhiza* tanshinones are not attributable to a single pathway but result from a synergistic restoration of intestinal homeostasis across multiple levels. The collective evidence, from the amelioration of pathological damage and inflammation to the reshaping of the gut microbiota and metabolome, converges into a coherent mechanism. This model posits that tanshinones act by first repairing the compromised intestinal barrier, which in turn facilitates the normalization of the gut microbial community. The remodeled microbiota then executes a beneficial reprogramming of the gut metabolome, rectifying deficits in SCFAs and anti-inflammatory lipids while suppressing harmful metabolites like specific bile acids. The final outcome of this cascade is the attenuation of the aberrant immune response and the resolution of inflammation. Thus, our work not only deciphers the mechanistic basis of a traditional remedy but also positions tanshinones as a promising multi-target therapeutic strategy for UC by targeting the critical microbiota-metabolite axis.

Overall, our study establishes that tanshinones ameliorate ulcerative colitis through a multi-faceted mechanism involving anti-inflammatory, barrier-protective, and microbiota-modulating effects. Crucially, multi-omics analysis revealed that the therapeutic outcome is orchestrated through a functional microbiota-metabolite axis, exemplified by the critical association between *Akkermansia* and immunomodulatory lipids like dihydroxyoctadecadienoic acid, and the reprogramming of key pathways such as bile acid biosynthesis. Our work thus provides a molecular mechanistic foundation for the traditional application of *Salvia miltiorrhiza* and highlights its potential as a novel microbiota-targeting therapeutic strategy for UC. Future studies should prioritize validating the causal relationships within this axis and exploring its clinical translatability.

## Supporting information

Supplementary_tabel

Supplementary_Table_S2

Supplementary_Table_S1

Supplementary_table

Supplementary_Material

Supplementary_Table_S3

## Abbreviations

The 16S rRNA amplicon sequencing data generated in this study have been deposited in the NCBI Sequence Read Archive (SRA) under the BioProject accession numbers PRJNA1423105 (for the effect of four tanshinones) and PRJNA1423154 (for the effect of Tan IIA + *M*. *Akk*). The data are publicly available at https://www.ncbi.nlm.nih.gov/bioproject/PRJNA1423105 and https://www.ncbi.nlm.nih.gov/bioproject/PRJNA1423154 upon publication. The raw LC-MS metabolomics data have been deposited in the MetaboLights database under the accession numbers MTBLS13897 (for the effect of four tanshinones) and MTBLS13899 (for the effect of Tan IIA + *M*. *Akk*). The data are accessible at https://www.ebi.ac.uk/metabolights/MTBLS13897 and https://www.ebi.ac.uk/metabolights/MTBLS13899 upon publication.

## Supplementary Data

Supplementary data are available at *Acta Biochimica et Biophysica Sinica* online.

## References

[1] Le Berre C, Honap S, Peyrin-Biroulet L (2023). Ulcerative colitis. Lancet.

[2] Wangchuk P, Yeshi K, Loukas A (2024). Ulcerative colitis: clinical biomarkers, therapeutic targets, and emerging treatments. Trends Pharmacol Sci.

[3] Neurath MF (2024). Strategies for targeting cytokines in inflammatory bowel disease. Nat Rev Immunol.

[4] Liang Y, Li Y, Lee C, Yu Z, Chen C, Liang C (2024). Ulcerative colitis: molecular insights and intervention therapy. Mol Biomed.

[5] Kaminsky LW, Al-Sadi R, Ma TY (2021). IL-1β and the intestinal epithelial tight junction barrier. Front Immunol.

[6] Ungaro R, Mehandru S, Allen PB, Peyrin-Biroulet L, Colombel JF (2017). Ulcerative colitis. Lancet.

[7] Morgan XC, Tickle TL, Sokol H, Gevers D, Devaney KL, Ward DV, Reyes JA (2012). Dysfunction of the intestinal microbiome in inflammatory bowel disease and treatment. Genome Biol.

[8] Schirmer M, Stražar M, Avila-Pacheco J, Rojas-Tapias DF, Brown EM, Temple E, Deik A (2024). Linking microbial genes to plasma and stool metabolites uncovers host-microbial interactions underlying ulcerative colitis disease course. Cell Host Microbe.

[9] Zhu S, Han M, Liu S, Fan L, Shi H, Li P (2022). Composition and diverse differences of intestinal microbiota in ulcerative colitis patients. Front Cell Infect Microbiol.

[10] Seishima J, Iida N, Kitamura K, Yutani M, Wang Z, Seki A, Yamashita T (2019). Gut-derived
*Enterococcus faecium* from ulcerative colitis patients promotes colitis in a genetically susceptible mouse host. Genome Biol.

[11] Čipčić Paljetak H, Barešić A, Panek M, Perić M, Matijašić M, Lojkić I, Barišić A (2022). Gut microbiota in mucosa and feces of newly diagnosed, treatment-naïve adult inflammatory bowel disease and irritable bowel syndrome patients. Gut Microbes.

[12] Machiels K, Joossens M, Sabino J, De Preter V, Arijs I, Eeckhaut V, Ballet V (2014). A decrease of the butyrate-producing species
*Roseburia hominis* and
*Faecalibacterium prausnitzii* defines dysbiosis in patients with ulcerative colitis. Gut.

[13] Wu R, Xiong R, Li Y, Chen J, Yan R (2023). Gut microbiome, metabolome, host immunity associated with inflammatory bowel disease and intervention of fecal microbiota transplantation. J Autoimmunity.

[14] Parada Venegas D, De la Fuente MK, Landskron G, González MJ, Quera R, Dijkstra G, Harmsen HJM (2019). Short chain fatty acids (SCFAs)-mediated gut epithelial and immune regulation and its relevance for inflammatory bowel diseases. Front Immunol.

[15] Hu Y, Chen Z, Xu C, Kan S, Chen D (2022). Disturbances of the gut microbiota and microbiota-derived metabolites in inflammatory bowel disease. Nutrients.

[16] Peng KY, Gu JF, Su SL, Zhu Y, Guo JM, Qian DW, Duan JA (2021). Salvia miltiorrhiza stems and leaves total phenolic acids combination with tanshinone protect against DSS-induced ulcerative colitis through inhibiting TLR4/PI3K/AKT/mTOR signaling pathway in mice. J EthnoPharmacol.

[17] Hu C, He X, Zhang H, Hu X, Liao L, Cai M, Lin Z (2024). Tanshinone I limits inflammasome activation of macrophage via docking into Syk to alleviate DSS-induced colitis in mice. Mol Immunol.

[18] Liu X, He H, Huang T, Lei Z, Liu F, An G, Wen T (2016). Tanshinone IIA protects against dextran sulfate sodium- (DSS-) induced colitis in mice by modulation of neutrophil infiltration and activation. Oxid Med Cell Longev.

[19] Min X, Zeng X, Zhao W, Han Z, Wang Y, Han Y, Pei L (2020). Cryptotanshinone protects dextran sulfate sodium-induced experimental ulcerative colitis in mice by inhibiting intestinal inflammation. PhytoTher Res.

[20] Guo Y, Wu X, Wu Q, Lu Y, Shi J, Chen X (2018). Dihydrotanshinone I, a natural product, ameliorates DSS-induced experimental ulcerative colitis in mice. Toxicol Appl Pharmacol.

[21] Liu J, Cai J, Fan P, Dong X, Zhang N, Tai J, Cao Y (2023). Salidroside alleviates dextran sulfate sodium-induced colitis in mice by modulating the gut microbiota. Food Funct.

[22] Lima SF, Pires S, Rupert A, Oguntunmibi S, Jin WB, Marderstein A, Funez-dePagnier G (2024). The gut microbiome regulates the clinical efficacy of sulfasalazine therapy for IBD-associated spondyloarthritis. Cell Rep Med.

[23] Nyström EEL, Martinez-Abad B, Arike L, Birchenough GMH, Nonnecke EB, Castillo PA, Svensson F (2021). An intercrypt subpopulation of goblet cells is essential for colonic mucus barrier function. Science.

[24] Zhang X, Zhang F, Li Y, Fan N, Zhao K, Zhang A, Kang J (2024). Blockade of PI3K/AKT signaling pathway by astragaloside IV attenuates ulcerative colitis via improving the intestinal epithelial barrier. J Transl Med.

[25] Gao H, Sun M, Li A, Gu Q, Kang D, Feng Z, Li X (2025). Microbiota-derived IPA alleviates intestinal mucosal inflammation through upregulating Th1/Th17 cell apoptosis in inflammatory bowel disease. Gut Microbes.

[26] Li G, Lin J, Zhang C, Gao H, Lu H, Gao X, Zhu R (2021). Microbiota metabolite butyrate constrains neutrophil functions and ameliorates mucosal inflammation in inflammatory bowel disease. Gut Microbes.

[27] Sasson AN, Ingram RJM, Zhang Z, Taylor LM, Ananthakrishnan AN, Kaplan GG, Ng SC (2021). The role of precision nutrition in the modulation of microbial composition and function in people with inflammatory bowel disease. Lancet Gastroenterol Hepatol.

[28] Yu Z, Li D, Sun H (2023). Herba Origani alleviated DSS-induced ulcerative colitis in mice through remolding gut microbiota to regulate bile acid and short-chain fatty acid metabolisms. Biomed Pharmacother.

[29] Vaga S, Lee S, Ji B, Andreasson A, Talley NJ, Agréus L, Bidkhori G (2020). Compositional and functional differences of the mucosal microbiota along the intestine of healthy individuals. Sci Rep.

[30] Liu TH, Chen GL, Lin CH, Tsai TY, Cheng MC (2025). *Lactobacillus plantarum* TWK10 relieves loperamide-induced constipation in rats fed a high-fat diet via modulating enteric neurotransmitters, short-chain fatty acids and gut microbiota. Food Funct.

[31] Gao B, Wang R, Peng Y, Li X (2018). Effects of a homogeneous polysaccharide from Sijunzi decoction on human intestinal microbes and short chain fatty acids
*in vitro*. J EthnoPharmacol.

[32] Park J, Kim NE, Yoon H, Shin CM, Kim N, Lee DH, Park JY (2021). Fecal microbiota and gut microbe-derived extracellular vesicles in colorectal cancer. Front Oncol.

[33] Gong X, Cai W, Yang D, Wang W, Che H, Li H (2025). Effect of the arabinogalactan from
*Ixeris chinensis* (Thunb.) Nakai. attenuates DSS-induced colitis and accompanying depression-like behavior. Int J Biol Macromol.

[34] Tajasuwan L, Kettawan A, Rungruang T, Wunjuntuk K, Prombutara P (2023). Role of dietary defatted rice bran in the modulation of gut microbiota in AOM/DSS-induced colitis-associated colorectal cancer rat model. Nutrients.

[35] Lavelle A, Sokol H (2020). Gut microbiota-derived metabolites as key actors in inflammatory bowel disease. Nat Rev Gastroenterol Hepatol.

[36] Attema B, Kuipers F (2025). Microbiome-derived secondary bile acids promote repair of colonic mucosa after injury. EMBO Mol Med.

[37] Jalil A, Perino A, Dong Y, Imbach J, Volet C, Vico-Oton E, Demagny H (2025). Bile acid 7α-dehydroxylating bacteria accelerate injury-induced mucosal healing in the colon. EMBO Mol Med.

[38] Zheng M, Han R, Yuan Y, Xing Y, Zhang W, Sun Z, Liu Y (2023). The role of
*Akkermansia muciniphila* in inflammatory bowel disease: current knowledge and perspectives. Front Immunol.

[39] Davey LE, Malkus PN, Villa M, Dolat L, Holmes ZC, Letourneau J, Ansaldo E (2023). A genetic system for
*Akkermansia muciniphila* reveals a role for mucin foraging in gut colonization and host sterol biosynthesis gene expression. Nat Microbiol.

[40] Bustamante JM, Dawson T, Loeffler C, Marfori Z, Marchesi JR, Mullish BH, Thompson CC (2022). Impact of fecal microbiota transplantation on gut bacterial bile acid metabolism in humans. Nutrients.

[41] Chang SC, Shen MH, Liu CY, Pu CM, Hu JM, Huang CJ (2020). A gut butyrate‑producing bacterium
*Butyricicoccus pullicaecorum* regulates short‑chain fatty acid transporter and receptor to reduce the progression of 1,2‑dimethylhydrazine‑associated colorectal cancer. Oncol Lett.

[42] Maruyama S, Matsuoka T, Hosomi K, Park J, Murakami H, Miyachi M, Kawashima H (2024). High barley intake in non-obese individuals is associated with high natto consumption and abundance of butyrate-producing bacteria in the gut: a cross-sectional study. Front Nutr.

