# Supplementary material for: Tanshinones from Salvia miltiorrhiza alleviate ulcerative colitis via reprogramming the gut microbiota-metabolite axis : Tanshinones alleviate UC via microbiota-metabolite axis
Source: Acta Biochim Biophys Sin (Shanghai). 2026 Mar 31;58(7):1579–96. doi: 10.3724/abbs.2026054 (PMC13449000; doi:10.3724/abbs.2026054)
Supplement: Supplementary file 1 [file Supplementary_Material.docx]

**Supplementary Material**

**Table of contents:**

**1. Supplementary Figure S1-S8**

**2. Supplementary Table S1-S5**

**Supplementary Figure S1. Tanshinones administration alleviate pathological manifestations of UC in mice** (A) Body weight changes of mice throughout the experimental period. (B) Statistical analysis of colon length. (C) Hematoxylin and eosin (H&E) staining for various organs or tissues (heart, liver, spleen, lung, and kidney). Scale bar: 200 μm. (D) Quantitative analysis of the positive staining area for occludin and mucin4. Data are presented as the mean±SD. Statistical significance was determined by one-way ANOVA with *post-hoc* test. **P*< 0.05, ***P*< 0.01, ****P*< 0.001, and ns means not significant.

**Supplementary Figure S2. Tanshinones administration mitigates inflammatory factor in DSS-induced colitis mice** (A‒D) The serum levels of inflammatory factors, including IL-1β, IL-4, IL-7, and TNF-α. Data are presented as the mean±SD. **P*<0.05, ****P*< 0.001 vs. Control group, ns, not significant. (E) Gene Ontology (GO) enrichment analysis of biological processes affected by tanshinones treatment.

**Supplementary Figure S3. Tanshinones reconstruct the gut microbiota composition in DSS-induced colitis mice** (A) Species accumulation curves indicating adequate sequencing depth for capturing microbial diversity. (B) Venn diagram showing the number of Amplicon Sequence Variants (ASVs) unique and common to each group. (C) Principal coordinates analysis (PCoA) plot based on weighted UniFrac distances, illustrating beta diversity. (D) Unsupervised Principal Component Analysis (PCA) plot further validating the separation of microbial communities. (E) Differences in predicted KEGG metabolic pathways among groups.

**Supplementary Figure S4. Network analysis of bacterial co-occurrence patterns** (A) Scatter plot of within-module connectivity (Zi) vs. among-module connectivity (Pi) for each node (genus). Nodes are categorized into four putative ecological roles based on their Zi and Pi values: peripherals (Zi ≤ 2.5, Pi ≤ 0.62), connectors (Zi ≤ 2.5, Pi > 0.62), module hubs (Zi > 2.5, Pi ≤ 0.62), and network hubs (Zi > 2.5, Pi > 0.62). Node color corresponds to bacterial phylum. (B) The degree distribution of the empirical network deviates from that of a random network, indicating a complex topological structure associated with microbial community stability. (C) A focused sub-network showcasing the interactions among the top 50 most abundant genera, highlighting the central hubs and key interactions that define the community structure

**Supplementary Figure S5. Multivariate statistical analysis and functional characterization of metabolic profiles** (A) Partial least squares-discriminant analysis (PLS-DA) score plot. (B) Orthogonal partial least squares-discriminant analysis (OPLS-DA) score plot. Both models demonstrate a clear separation of metabolic phenotypes among the Control, Model, and treatment groups. (C) Volcano plot of differentially abundant metabolites. Metabolites meeting both variable importance in projection (VIP) > 1.0 from the PLS-DA model and *P*-value < 0.05 (Student’s *t*-test) criteria are highlighted. (D) Hierarchical clustering heatmap of significantly altered metabolites (VIP > 1.0, *P* < 0.05). Rows represent metabolites, and columns represent individual samples. The color gradient from blue to red corresponds to low to high scaled intensity, illustrating distinct metabolite expression patterns across groups. (E) Sensitivity analysis of enriched KEGG pathways based on the Outcome-Direct Confounding (ODC) impact metric, evaluating the robustness of the mediation effect against potential confounding.

**Supplementary Figure S6. The effect of tanshinones on the growth of *Akkermansia muciniphila (M.Akk)*** (A) Growth curve of *M.Akk* over a 96-h period. (B) Direct effect of four tanshinone compounds at varying concentrations on *M.Akk* growth *in vitro*. Data are presented as the mean±SD (*n* = 3 independent cultures).

**Supplementary Figure S7. TanIIA and *Akkermansia muciniphila* synergistically ameliorate colitis by restoring the gut mucosal barrier and microbiota homeostasis** (A) Colon length measurement across experimental groups. (B) Representative H&E staining illustrating colonic pathological changes. (C) Immunohistochemical staining of mucosal barrier-related proteins (ZO-1 and mucin1). (D-G) Quantitative analysis of mucosal barrier protein expression: ZO-1 (D), mucin1 (E), mucin4 (F), and occludin (G). Data are presented as the mean±SD; **P* < 0.05, ***P* < 0.01, and ****P*< 0.001. (H) Relative abundance of gut microbiota at the genus level. (I) Functional prediction of gut microbiota via PICRUSt2 (KEGG database) reveals treatment-modulated metabolic pathways.

**Supplementary Figure S8. Fecal metabolomic profiling reveals therapeutic remodeling by TanIIA and *Akkermansia muciniphila* in colitis**	(A) Heatmap showing the relative abundance of the top 50 differentially abundant metabolites across experimental groups. (B) Bar plot of the inferred relative abundance of KEGG pathways (Level 2) based on PICRUSt2 prediction for the 331 differential metabolites (165 upregulated, 166 downregulated) identified from the TanIIA+*M.Akk*+DSS versus TanIIA+*M.Akk* comparison. (C) KEGG pathway enrichment analysis of differential metabolites identified from the TanIIA+*M.Akk*+DSS versus TanIIA+*M.Akk* comparison. Significantly enriched pathways are displayed, with point size representing the number of metabolites mapped to each pathway and color indicating the enrichment significance.
